# P-599. 6-Valent, OspA-Based VLA15 Lyme Disease Vaccine Candidate Against Lyme Borreliosis in a Healthy Pediatric and Adult Study Population: A Phase 2 Study Update

**DOI:** 10.1093/ofid/ofae631.797

**Published:** 2025-01-29

**Authors:** Marc D Messier, Laura Wagner, Romana Hochreiter, Erik Lamberth, Julian Larcher-Senn, Raphael Simon, James groark, James H Stark

**Affiliations:** VALNEVA GMBH, Lyon, Rhone-Alpes, France; Valneva Austria GmbH, Wien, Wien, Austria; Valneva GMBH, Vienna, Wien, Austria; Pfizer Inc, Collegeville, Pennsylvania; Assign Data Management and Biostatistics GmbH, Innsbruck, Tirol, Austria; Pfizer, New York, New York; Pfizer, New York, New York; Pfizer Biopharma Group, Collegeville, Pennsylvania

## Abstract

**Background:**

Lyme borreliosis (LB) is the most common tick-borne disease in the US, with approximately 476,000 cases diagnosed and treated annually and nearly 90 million individuals living in high incidence jurisdictions. Vector-focused prevention measures have varying levels of effectiveness due to low utilization, poor adherence, or high cost. A safe and efficacious vaccine to prevent LB is warranted. We provide an update on the Pfizer-Valneva VLA15 vaccine clinical development program, specifically the safety and immunogenicity booster data of the phase 2 trial (NCT04801420).

**Methods:**

A randomized, observer-blinded, multi-centre Phase 2 study investigated the safety and immunogenicity of VLA15 in adult and pediatric study populations. A total of 625 participants aged 5-65 years were enrolled in three age groups (5-11 years, 12-17 years, and 18-65 years) and randomized 1:1:1 to receive VLA15 180 mcg with alum in a three-dose (Month 0-2-6) or a two-dose schedule (Month 0-6), or three injections of placebo (Month 0-2-6) followed by booster doses at Month 18 and Month 30. Safety and immunogenicity data up to one month after completion of the primary vaccination series and yearly booster doses are presented.

**Results:**

Up to Month 19, VLA15 was safe and well tolerated in all age groups after all doses. Most adverse reactions were mild or moderate in severity. No related SAEs or other safety concerns were reported. The VLA15 booster dose induced a robust, anamnestic immune response against all six OspA vaccine serotypes in all age groups. Immune responses in children were higher than in adults across both schedules.

**Conclusion:**

The Month 18 VLA15-221 booster dose was safe and immunogenic in both adults and children. The Month 30 booster data is under analysis. The ongoing blinded Phase 3 trials in Europe and North America will provide additional data which may support use of this vaccine as the primary preventative measure to protect against LB.

**Disclosures:**

**Marc D. Messier, PhD**, SOBI: Advisor/Consultant|Valneva: employee|Valneva: Ownership Interest **Laura Wagner, M.Sc.**, Valneva: employee **Romana Hochreiter, PhD**, Valneva Austria GmbH: WO2021/207615 A1|Valneva Austria GmbH: Employee, owner of stock options **Erik Lamberth, MD**, Pfizer: Employee|Pfizer: Stocks/Bonds (Public Company) **Julian Larcher-Senn, PhD**, Valneva: Advisor/Consultant **Raphael Simon, PhD**, Pfizer: Stocks/Bonds (Private Company) **James groark, MD**, Pfizer: Stocks/Bonds (Public Company) **James H. Stark, PhD**, Pfizer: Employee|Pfizer: Stocks/Bonds (Public Company)

